# Upregulation of CREM/ICER suppresses wound endothelial CRE-HIF-1α-VEGF-dependent signaling and impairs angiogenesis in type 2 diabetes

**DOI:** 10.1242/dmm.017145

**Published:** 2014-11-07

**Authors:** Milad S. Bitar, Fahd Al-Mulla

**Affiliations:** 1 Department of Pharmacology and Toxicology, Kuwait University, Safat 13110, Kuwait.; 2 Department of Pathology, Kuwait University, Safat 13110, Kuwait.

**Keywords:** CREM/ICER, Angiogenesis, Diabetes, cAMP, VEGF

## Abstract

Impaired angiogenesis and endothelial dysfunction in type 2 diabetes constitute dominant risk factors for non-healing wounds and most forms of cardiovascular disease. We propose that diabetes shifts the ‘angiogenic balance’ in favor of an excessive anti-angiogenic phenotype. Herein, we report that diabetes impairs *in vivo* sponge angiogenic capacity by decreasing VEGF expression and fibrovascular invasion, and reciprocally enhances the formation of angiostatic molecules, such as thrombospondins, NFκB and FasL. Defective *in vivo* angiogenesis prompted cellular studies in cultured endothelial cells derived from subcutaneous sponge implants (SIECs) of control and Goto-Kakizaki rats. Ensuing data from diabetic SIECs demonstrated a marked upregulation in cAMP-PKA-CREB signaling, possibly stemming from increased expression of adenylyl cyclase isoforms 3 and 8, and decreased expression of PDE3. Mechanistically, we found that oxidative stress and PKA activation in diabetes enhanced CREM/ICER expression. This reduces IRS2 cellular content by inhibiting cAMP response element (CRE) transcriptional activity. Consequently, a decrease in the activity of Akt-mTOR ensued with a concomitant reduction in the total and nuclear protein levels of HIF-1α. Limiting HIF-1α availability for the specific hypoxia response elements in diabetic SIECs elicited a marked reduction in VEGF expression, both at the mRNA and protein levels. These molecular abnormalities were illustrated functionally by a defect in various pro-angiogenic properties, including cell proliferation, migration and tube formation. A genetic-based strategy in diabetic SIECs using siRNAs against CREM/ICER significantly augmented the PKA-dependent VEGF expression. To this end, the current data identify the importance of CREM/ICER as a negative regulator of endothelial function and establish a link between CREM/ICER overexpression and impaired angiogenesis during the course of diabetes. Moreover, it could also point to CREM/ICER as a potential therapeutic target in the treatment of pathological angiogenesis.

## INTRODUCTION

Angiogenesis, the sprouting of new capillaries from pre-existing vasculature, is a key step in several physiological and pathophysiological settings – including wound healing, cancer, neovascular disease of the eye and ischemia of the heart, the brain and the limbs. To proceed normally, angiogenesis requires the degradation of extracellular matrix proteins, as well as endothelial cell proliferation, migration and tube formation (Carmeliet, 2005). These steps are controlled by a number of pro-angiogenic (e.g. vascular endothelial growth factor, VEGF) and anti-angiogenic (thrombospondin, TSP; decorin) factors and involve several intracellular signaling pathways, including cAMP, nitric oxide (NO), mitogen activated kinases (MAPKs), phosphoinositide-3 kinase (PI3K)-Akt, PLC-γ and FAK-paxillin (Fukumura et al., 2001; Hood et al., 2003; Lee et al., 2006). In this context, adenylyl cyclase (AC)-inducing agents, such as prostaglandin E2 (PGE2), norepinephrine (NE) and forskolin appear to promote angiogenesis through a mechanism involving both the activation of cAMP-dependent protein kinase A (PKA) and the newly recognized family of cAMP-binding proteins named exchange protein directly activated by cAMP (EPAC) (Namkoong et al., 2009; Park et al., 2011; Zhang and Daaka, 2011). As a result, cAMP response element binding protein (CREB), Akt and endothelial nitric oxide synthase (eNOS) are phosphorylated, concomitant with the production of NO and VEGF (Bir et al., 2012; Namkoong et al., 2009; Syed-Abdul et al., 2011; Zhang and Daaka, 2011). By contrast, a diminution in angiogenic capacity is evident in response to PKA inhibition, TSPs or decorin induction (Kyriakides and Maclauchlan, 2009; Neill et al., 2012).

Type 2 diabetes has emerged as a major threat to human health, and it is considered to be a dominant risk factor for most forms of cardiovascular disease and non-healing wounds. In line with this, the incidence of stroke, claudication and myocardial ischemia or infarction has been reported to increase during the course of diabetes (Peters et al., 2014). Moreover, after acute limb ischemia or foot ulcers, diabetics appear to have both higher mortality and an increased rate of amputation (Jeffcoate and Harding, 2003). Altered angiogenesis and endothelial dysfunction are common features of type 2 diabetes, and they might contribute to some of the aforementioned abnormalities (Bitar et al., 2010; Bitar et al., 2005; Tahergorabi and Khazaei, 2012). This notion is consistent with several lines of evidence that support the view that angiogenesis is an integral part of the endogenous tissue repair mechanism that occurs after ischemic injury and in response to incisional and excisional wounds (Lähteenvuo and Rosenzweig, 2012). To this end, delineating the signaling cascades responsible for diabetes-related impairment of angiogenesis and endothelial function should have important implications for understanding and treating the various forms of cardiovascular disease and non-healing wound.

In view of the above information and our previous findings demonstrating that NO bioavailability is diminished (Bitar et al., 2005) and fibrovascular invasion is reduced as a function of diabetes (Bitar, 1998; Bitar, 2000), a hypothesis was articulated stating that a signaling axis through PKA, CREB and hypoxia-inducible factor 1α (HIF-1α) (a powerful activator of VEGF-mediated angiogenesis) is altered during the course of diabetes. As an initial step toward supporting this notion, we investigated in Goto-Kakizaki rats (GK rats, a model for non-obese type 2 diabetes) the possible involvement of PKA and its downstream effectors in angiogenesis by using the *in vitro* endothelial cell cultures and *in vivo* sponge implant angiogenic assays. Here, we newly identify cAMP response element modulator [CREM, also known as inducible cAMP early repressor (ICER)] as a signaling player that links the defect in PKA- and CREB-mediated VEGF production to impaired angiogenesis during the course of diabetes. Indeed, normalization of CREM/ICER overexpression in diabetic endothelial cells using a genetic-based strategy, exemplified through use of a small interference (si)RNA against ICER, appears to correlate with a significant elevation of VEGF both at the mRNA and protein levels.

**TRANSLATIONAL IMPACT**

**Clinical issue**

Diabetes is the predominant risk factor for cardiovascular diseases and non-healing wounds. Angiogenesis, the production of new blood vessels from pre-existing ones, is an essential adaptive response in tissue healing and ischemic injury. This phenomenon is controlled by the levels and distribution of both pro-angiogenic (e.g. vascular endothelial growth factor, VEGF) and anti-angiogenic (e.g. thrombospondins, TSPs) factors and appears to be impaired in selective tissues during the course of type 2 diabetes. To this end, examining the basis of diabetes-mediated impairment of angiogenesis and endothelial function is likely to offer a better understanding of the pathogenesis and treatment of the various forms of cardiovascular disease and non-healing wounds.

**Results**

Here, the authors studied the angiogenic network by using *in vivo* (subcutaneous) and *in vitro* sponge implants of endothelial cells (SIECs) from diabetic rats as models of angiogenesis in type 2 diabetes. Results showed that the expression level of VEGF was decreased, whereas that of TSPs was increased in these models as compared to control tissues. The decrease in VEGF level as a function of diabetes appears to be associated with a significant attenuation in the transcriptional activities of cAMP response element (CRE) and hypoxia-inducible factor 1α response element (HRE). A triggering event for these changes was identified to reflect the overexpression of cAMP response element modulator (CREM/ICER). Indeed, reducing the level of expression of CREM/ICER using an siRNA-based strategy ameliorated diabetes-related suppression of *VEGF* promoter activity.

**Implication and future direction**

The finding that CREM/ICER are involved in reduced VEGF levels and, possibly, impaired angiogenesis during the course of type 2 diabetes is likely to have crucial implications for understanding the pathogenesis of endothelial dysfunction. Moreover, the molecular changes uncovered in this study might open up new avenues for therapeutic interventions that target individuals with tissue ischemia and non-healing wounds. Future clinical studies should provide more in-depth evidence-based support for considering CREM/ICER inhibitors and activators for the treatment of pathological angiogenesis.

## RESULTS

### Diabetes-induced impairment of angiogenic capacity in subcutaneous sponge implants

**Fig. 1.**
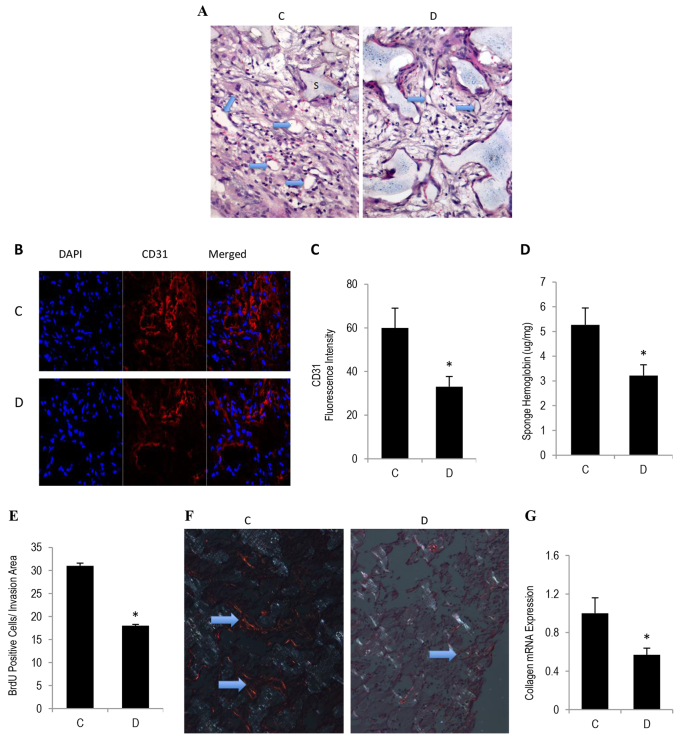
**Diabetes-induced impairment in angiogenic capacity in subcutaneous sponge implants.** Sterile sponge discs were implanted subcutaneously into control and GK diabetic rats that had been anesthetized with ketamine and xylazine. Sponges were retrieved 10–12 days post-implantation, bisected and either embedded and sectioned or frozen in liquid nitrogen and used for microscopic or biochemical analysis of angiogenic capacity. (A) Representative photomicrographs of hematoxylin-eosin-stained sponge sections revealing fibrovascular invasion. Arrows denote the presence of large and small blood vessels. (B) Representative photomicrographs of CD31 immunofluorescence staining. (C) Quantitation of CD31 fluorescence intensity from images shown in B. (D) Hemoglobin content in the sponges, as determined by using the Drabkin reagent. (E) A proliferation response in sponge implants was assessed by immunostaining for BrdU. (F) Picrosirius-Red-stained sponge sections were viewed under a polarized-light microscope and revealed the presence of red tightly packed mature collagen fibers in sponges from control mice, but these were present to a much lesser extent in sponges from diabetic mice. (G) Collagen type 1 mRNA content from sponges, as assessed by qRT-PCR analyses. ‘C’, control; ‘D’, diabetic. Results are expressed as means±s.e.m. from three independent experiments. *Significantly different from corresponding control values at *P*≤0.05.

To evaluate the diabetic-state effect on angiogenic capacity *in vivo*, sponge implants were inserted subcutaneously into control and GK rats. This model provides scaffolding for dividing endothelial cells and, unlike Matrigel, the sponge implants maintain their shape and size for up to 4 weeks. During the early phase (1–2 weeks), cell invasion into the sponge and new tissue formation permit the quantification of neovascularization. Hematoxylin and eosin staining revealed that sponges that had been implanted into type 2 diabetic mice exhibited a decrease in the degree of fibrovascular invasion (Fig. 1A). It is noteworthy that the sponges from diabetic mice appeared to have a bigger space than corresponding controls, a phenomenon which might stem, at least in part, from an increased derivative capacity in this disease state. Consistent with these data, we also confirmed, by using immunofluorescence microscopy and a spectrophotometry-based technique, a reduction in the CD31 (Fig. 1B,C) and hemoglobin (Fig. 1D) content of sponges from diabetic mice, indicating that the processes involved in capillary formation in connection with blood vessel function are attenuated during the course of diabetes. Large blood vessels, as indicated by arrows in the figure, predominate in sponge sections from control but not in those from diabetic mice (Fig. 1A). Because proliferation, like that of migration, constitutes an essential element of the angiogenic network, we assessed this process by determining the rate of bromodeoxyuridine (BrdU) incorporation into cell nuclei. Approximately 17% of the cells appeared to be proliferating, and Fig. 1E shows that the absolute number of cells that stained positively for BrdU, corrected for the percentage area of fibrovascular invasion, was less in sponges of type 2 diabetes, as compared to corresponding control values.

Next, we determined in terms of quality and mRNA the status of collagen deposition in sponge implants during the course of angiogenesis. Sirius-Red-stained sections that were visualized under a polarized microscope confirmed that the level of red-stained tightly packed mature collagen appeared to be decreased as a function of diabetes (Fig. 1F). Similarly, quantitative real-time (qRT)-PCR revealed that the mRNA level of type 1 collagen was also reduced in this disease state (Fig. 1G). Collectively, the above data suggest that neoangiogenesis is impaired in a sponge model of type 2 diabetes. This phenomenon (e.g. impaired angiogenesis) might provide a partial explanation for the increased prevalence of both cardiovascular diseases and non-healing wounds during the course of diabetes.

### Diabetes represses angiogenesis by increasing apoptosis and by altering a tightly regulated balance between sponge implants and angiogenic inducers and inhibitors

**Fig. 2.**
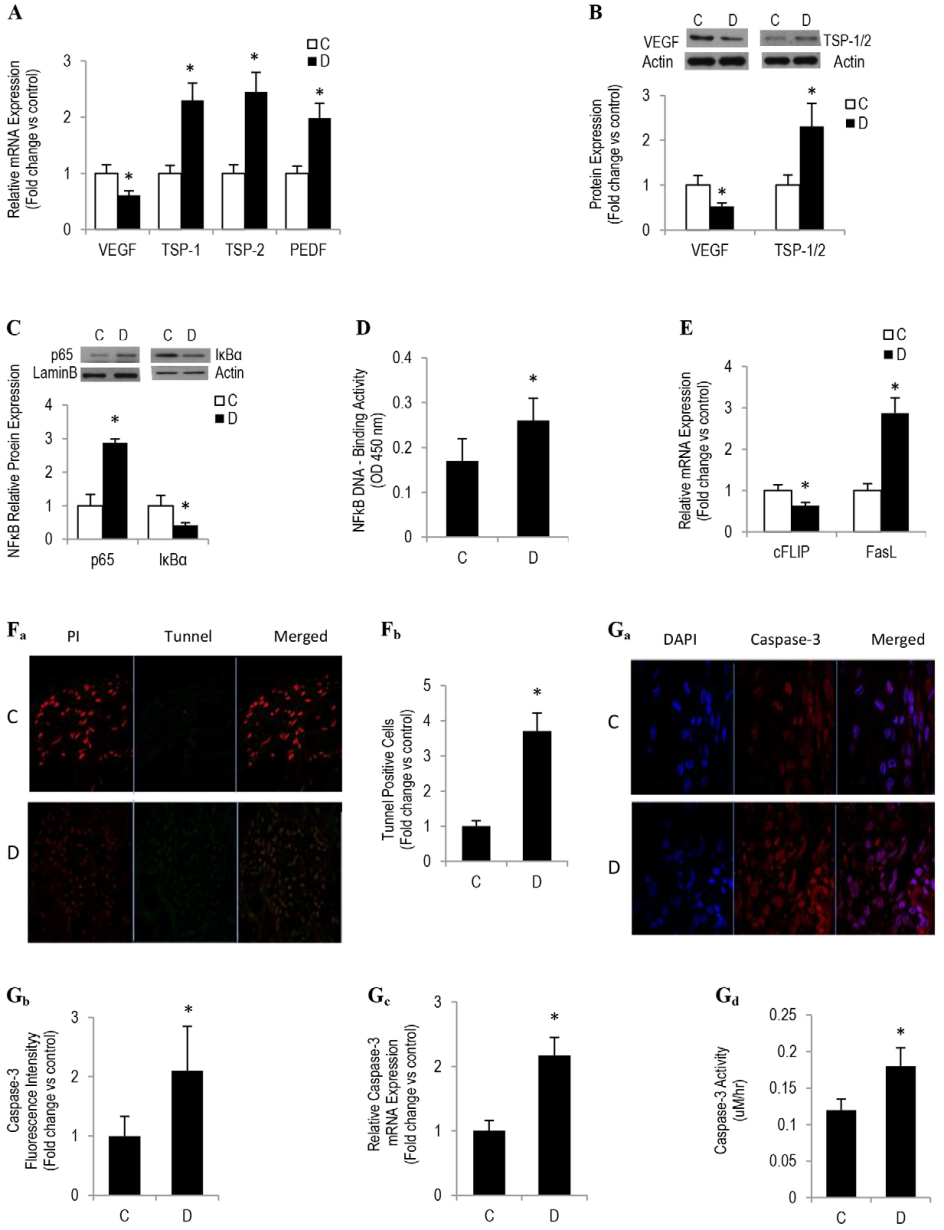
**Diabetes inhibits angiogenesis by increasing apoptosis and attenuating pro-angiogenic factors.** qRT-PCR analyses measuring mRNA expression of *VEGF*, *TSP1*, *TSP2* and *PEDF*. (B) Western blot analysis demonstrating protein expression of *VEGF*, TSP1 and TSP2. The graph below shows quantitation of the blot as the fold change relative to the control. (C) A representative image of a western blot revealing p65 nuclear localization. The graph below shows the quantitative measure of fold change in nuclear p65 level. (D) TransAM NFκB transcription factor ELISA-based assay showing the degree of p65 binding to nuclear extracts derived from sponge implants from control and diabetic mice. (E) qRT-PCR analyses measuring the mRNA expression of *cFLIP* and *FasL*. (Fa) Representative immunofluorescence images confirming the presence of TUNEL-positive cells. (Fb) Quantitative measure of the fold change in the intensity of green-stained cells. (Ga) Immunofluorescence images demonstrating the presence of cleaved caspase 3. (Gb) Quantitative measure of the fold change in the intensity of the fluorescence of the staining of caspase 3. (Gc) qRT-PCR analyses of the expression of caspase 3 mRNA. (Gd) A fluorometric-based assay assessing the basal activity of caspase 3. ‘C’, control; ‘D’, diabetic. Results are expressed as means±s.e.m. from three independent experiments. *Significantly different from corresponding control values at *P*≤0.05.

Equilibrium between endogenous anti-angiogenic and pro-angiogenic molecules maintains the ‘angiogenic balance’, whereas in conditions such as diabetes, we propose that this balance is shifted in favor of an anti-angiogenic phenotype, henceforth contributing, at least in part, to impaired angiogenesis and delayed wound healing. To investigate this connection, we assessed the mRNA and protein expression of the most potent angiogenic inducers (e.g. VEGF) and inhibitors [e.g. TSP1 and TSP2, as well as pigment epithelial-derived factor (PEDF)] in the sponge implants using a combination of approaches, which included techniques based on real-time PCR, western blotting, ELISA and immunofluorescence. Quantitative real-time PCR studies revealed that mRNA levels for *VEGF* were decreased, whereas those for TSP1, TSP2 and PEDF were increased in sponges retrieved from GK diabetic rats (Fig. 2A). Consistently, this inverse relationship between *VEGF* and *TSP* mRNA expression as a function of diabetes was recapitulated at the protein levels by using a western-blotting-based technique (Fig. 2B).

TSPs and PEDF appeared to reduce angiogenic capacity, not only by inhibiting the activity of VEGF but also through a mechanism involving an increase in nuclear factor kappa B (NF-κB)-DNA binding activity (Aurora et al., 2010; Lawler and Lawler, 2012). In this context, an induction of NF-κB through anti-angiogenic molecules (e.g. TSPs and PEDF) suppresses the anti-apoptotic, pro-angiogenic protein cFLIP and activates the pro-apoptotic, anti-angiogenic FasL (Aurora et al., 2010; Chan et al., 1999). To this end, we sought to examine whether NF-κB-cFLIP-FasL-dependent signaling is altered diabetic angiogenic milieu, in which TSPs and PEDF are hyperactive.

An initial assessment of NF-κB dynamics using western blotting analysis revealed an enhancement in nuclear p65 content and a modest decrease in the total levels of the NF-κB inhibitor IκBα in the sponge implants from type 2 diabetic mice (Fig. 2C). Consistent with these data, a promoter-based ELISA assay also confirmed a significant increase in the DNA binding of NF-κB p65 in this disease state (Fig. 2D).

Next, we questioned whether a diabetes-induced increase in TSP- or PEDF-NF-κB-dependent pathways in sponges has an impact on key apoptotic or survival targets during the course of angiogenesis. Quantitative PCR analysis demonstrated a diminution in *cFLIP* and an augmentation in *FasL* mRNA levels in sponges from mice with type 2 diabetes (Fig. 2E). To identify and quantify the presence of apoptosis, we performed tunnel labeling in sponge sections from control and diabetic rats. Representative images showed a few isolated TUNEL-positive green fluorescent cells in control sections, whereas a large number of these cells were seen in sections derived from GK diabetic rats (Fig. 2Fa). Quantitation of TUNEL-positive cells revealed about a fourfold increase of apoptotic cells in diabetic sections when compared with corresponding control values (Fig. 2Fb). To confirm these findings, we evaluated the expression and degree of activation of caspase 3, a key executioner enzyme in the program of apoptosis, by using *in situ* immunofluorescence, real-time PCR and fluorometric enzyme activity assays. The data demonstrated that the level of immunoreactive product for cleaved caspase 3 (Fig. 2Ga,b), the degree of expression of mRNA encoding caspase 3 (Fig. 2Gc) and, finally, the activity of this executioner enzyme of the apoptotic program (Fig. 2Gd) were all dramatically increased as a function of diabetes.

Overall, the above data taken together support the notion that impaired sponge angiogenic capacity during the course of diabetes might stem, at least in part, from a combination of decreased VEGF and/or cFLIP expression and increased activity of TSP- or PEDF-NF-κB-FasL-dependent signaling. This reciprocal change between angiostatic molecules and pro-angiogenic gene targets could elicit a non-permissive anti-angiogenic microenvironment that has the characteristic features of decreased rate of growth and a heightened level of apoptotic cell death. The upstream signaling cascade responsible for diabetes-related suppression of VEGF expression is to be elaborated upon in the next few sections, whereas the mechanistic studies involving the upregulation of TSPs are currently under consideration in our laboratory.

### Impaired angiogenesis in type 2 diabetes might stem from altered PKA-CREB-CRE-dependent signaling

To explore the mechanism underpinning our *in vivo* findings of impaired angiogenesis and decreased VEGF levels, we investigated, in cultured endothelial cells that had been derived from control and diabetic sponge implants (SIECs), cAMP-PKA-dependent signaling under basal conditions and in response to the PKA activator N6-monobutyl-cAMP (MB-cAMP). This strategy is reasonable because a number of adenylyl-cyclase-activating agents, such as forskolin and PGE2, have been shown to enhance the generation of pro-angiogenic inducers, including VEGF and NO (Namkoong et al., 2009; Zhang and Daaka, 2011). Moreover, they also promote the *in vitro* tube formation of human microvascular endothelial cells, *ex vivo* vessel outgrowth of aortic rings and actual *in vivo* angiogenesis (Namkoong et al., 2009; Zhang and Daaka, 2011).

**Fig. 3.**
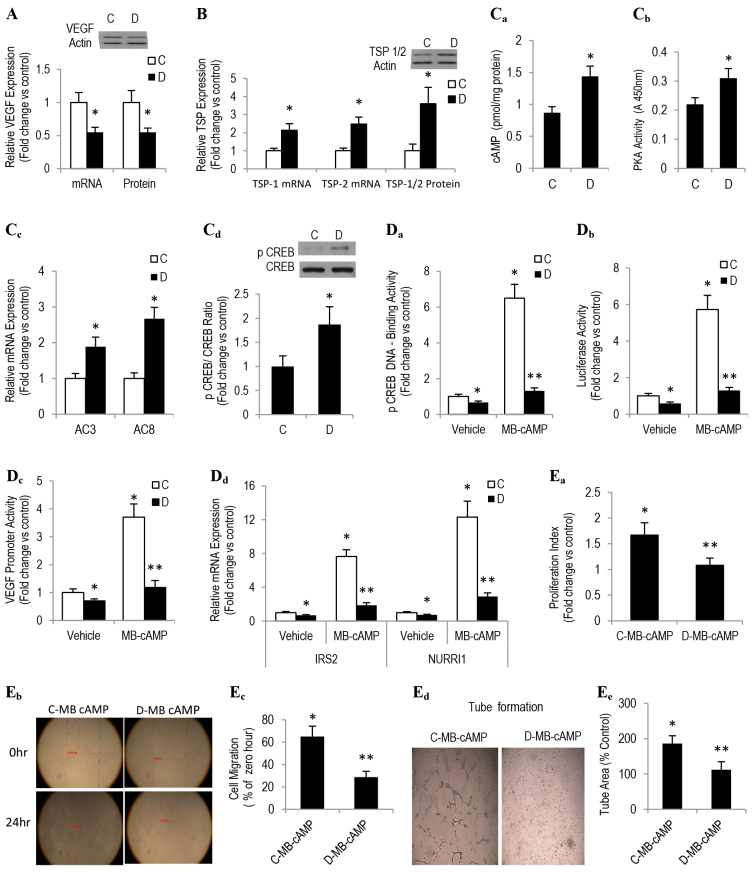
**Diabetes antagonizes angiogenesis by altering PKA-CREB-VEGF-dependent signaling.** Endothelial cells isolated from sponge implants (SIECs) from control and diabetic mice were used to assess angiogenic capacity and the cAMP-PKA-dependent pathway. qRT-PCR- and western-blotting-based techniques were used in the determination of mRNA (lower graph) and protein levels (upper blot), respectively, of (A) VEGF and (B) TSP1 and TSP2. (Ca) cAMP level was measured using ELISA, (Cb) PKA activity was measured using enzyme activity and (Cc) *AC3* and *AC8* mRNA expression was measured qRT-PCR. (Cd) Similarly, total cellular contents of p-CREB were assessed by western blot analysis. (Da) pCREB-DNA binding activity in nuclear extracts was assessed by a TransAM-ELISA-based assay. (Db,c) SIECs were transiently transfected with the luciferase reporter plasmid containing the (Db) CRE or (Dc) *VEGF* promoter and then treated with 100 μM MB-cAMP for 6 hours (CRE) or 12 hours (VEGF), and the relative luciferase activity in cell extracts was measured with a luminometer. (Dd) The mRNA expression of a number of CREB target genes containing a conserved CRE was determined by using real-time PCR analyses. Angiogenic capacity was evaluated in terms of (Ea) cell proliferation (e.g. 24-hour starved cells were treated with MB-cAMP for 24 hours in the presence of 10 μM BrdU followed by fixation and assaying the rate of BrdU incorporation into DNA; (Eb,c) cell migration (e.g. scratching 24-hour starved cells with a pipette tip followed by measuring the percentage of the wound covered by cells under a light microscope; Eb shows example images, Ec shows the quantitation) and (Ed,e) tube formation (e.g. serum-starved cells were seeded on growth-factor-reduced Matrigel. Ed shows example photographs that were taken after 24 hours, Ee shows the quantitation). ‘C’, control; ‘D’, diabetic. Results are expressed as means±s.e.m. from three independent experiments. *Significantly different from corresponding vehicle-treated control values at *P*≤0.05. **Significantly different from corresponding MB-cAMP-treated control values at *P*≤0.05.

As an initial step toward the above notion, we confirmed that diabetic SIECs in culture exhibited multiple angiogenic phenotypic features that were reminiscent of those documented in the *in vivo* sponge implants retrieved from GK rats with type 2 diabetes. Indeed, our data showed that VEGF expression both at the mRNA and protein level was diminished in diabetic SIECs, as compared with corresponding control values (Fig. 3A). By contrast, an upregulation in the production of TSPs was evident in these cells (Fig. 3B).

Realizing the importance of the cAMP-PKA-dependent pathway in regulating the angiogenic network, we questioned whether it contributes to impaired angiogenesis during the course of diabetes. Fig. 3Ca,b shows that PKA activity and the level of cAMP were increase in diabetic SIECs, as compared with those of corresponding control SIECs. This was associated with a similar enhancement in mRNA levels of the different isoforms of adenylyl cyclase, including AC3 and AC8 (Fig. 3Cc). Consistent with these findings, a significant elevation in the cellular contents of phosphorylated CREB (pCREB) was also evident as a function of diabetes (Fig. 3Cd). To confirm that the above diabetes-induced elevation in pCREB was mediated by PKA, and not other kinases known to activate CREB, the expression levels of Ca^2+^-calmodulin kinase (CAMK), p44/42 MAPK and p90 ribosomal S6 kinase were examined and found to be unaltered, or even decreased, in diabetic SIECs (data not shown).

These results are unexpected, especially when viewed in the context of the current data depicted in Fig. 3A and those reported previously, because they demonstrate that activation of CREB induced, whereas inhibition of CREB blocked, VEGF expression in various cell lines (Namkoong et al., 2009; Wu et al., 2007). Rectifying such ambiguity dictated the assessment of pCREB binding to the cAMP response element (CRE) in endothelial nuclear extracts under basal conditions and in response to MB-cAMP using the Tran-AM-ELISA-based assay. The data derived from these studies revealed a marked reduction in the amount of pCREB that was bound to CRE oligonucleotides in SIECs of type 2 diabetes (Fig. 3Da). Treatment with MB-cAMP increased the association of pCREB with CRE by about sixfold in control SIECs, a phenomenon which was attenuated as a function of diabetes (Fig. 3Da). Consistent with these findings, the relative CRE-, and more importantly, VEGF-luciferase reporter gene activities, a functional measure of the CREB-DNA binding potential, were also reduced in this disease state (Fig. 3Db,c).

As a surrogate for CRE transcriptional activity, we quantified in diabetic SIECs the level of mRNA expression of a number of CREB target genes that contain a conserved CRE (e.g. insulin receptor substrate 2, IRS2; unclear orphan receptor, NURR1/NR4A2) by using qRT-PCR analyses, corrected for the transcript expression of 18S, and found these RNAs to be downregulated both under basal conditions and also in response to 2 hours of treatment with MB-cAMP (Fig. 3Dd). The most intriguing findings, however, are the data depicted in Fig. 3Ea–e, which demonstrate a diminution in the proliferation (Fig. 3E,a), migration (Fig. 3Eb,c) and tube formation (Fig. 3Ed,e) of SIECs in response to MB-cAMP during the course of diabetes. To this end, the current data support the premise that pCREB-CRE binding is attenuated and that this might contribute to reduced VEGF levels and impaired angiogenesis in diabetic SIECs.

### Diabetes-induced impairment in pCREB-CRE binding activity alters HIF-1α dynamics and contributes to reduced VEGF expression in SIECs

**Fig. 4.**
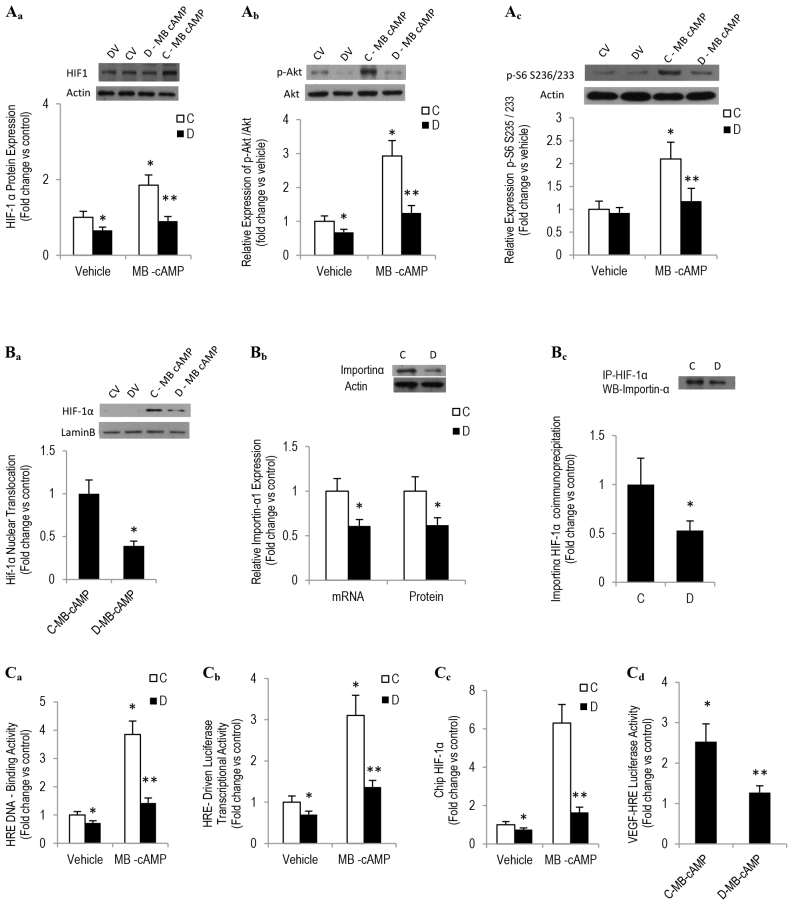
**Altered HIF-1α dynamics contribute to reduced VEGF expression in diabetic SIECs.** (Aa–c) Total cell contents of HIF-1α, pAkt (at residue S473) and pS6 (at residues S235/236) were assessed in SIECs that had been exposed to MB-cAMP for 16 hours. Example blots are shown at the top, quantitation of the blots is shown in the graphs below. (Ba) HIF-1α nuclear localization in SIECs treated with MB-cAMP for 2 hours was determined using a cell fractionation kit (Active Motif). A representative blot is shown, and quantitation of the blot is shown underneath. (Bb) Similarly, importin α mRNA and protein expression in SIECS was assessed using qRT-PCR (graph) and western blotting (blot). (Bc) The binding affinity of HIF-1α for importin α was evaluated by using co-immunoprecipitation followed by western blotting. The graph shows the quantitation of the blot. (Ca) The transcription factor ELISA assay revealed that MB-cAMP (100 μM) elicited the specific binding of SIEC nuclear extracts to the HRE consensus in a time-dependent manner, which was inhibited by wild-type but not mutated HRE oligonucleotide. (Cb) SIECs were transfected with a HRE-driven luciferase reporter construct and *Renilla* luciferase control plasmid for 24 hours. After transfection, cells were exposed to MB-cAMP (100 μM) for 12 hours and the intensity of luciferase reactions normalized to their *Renilla* luciferase control activity was measured using a dual luciferase assay kit. (Cc) A ChIP assay showed an interaction of HIF-1α with the HRE-containing promoter region of *VEGF* in SIECs that had been treated with MB-cAMP (100 μM) for 4 hours. (Cd) SIECs were transiently transfected with a plasmid expressing the reporter gene luciferase under the control of a fragment of the *VEGF* prompter containing the HRE, and then the intensity of the luciferase activity was measured 24 hours following MB-cAMP exposure. ‘C’, control; ‘D’, diabetic. Results are expressed as means±s.e.m. for three independent experiments. *Significantly different from corresponding control values at *P*≤0.05. **Significantly different from corresponding MB-cAMP-treated control values at *P*≤0.05.

A panoply of evidence indicates that efficient binding of pCREB binding to CRE recruits HIF-1α, an oxygen-sensing heterodimer and a major regulator of VEGF expression, and facilitates the binding of HIF-1α to a hypoxia response element (HRE) within the promoter region of *VEGF* (Wu et al., 2007). Moreover, it might also enhance HIF-1α protein accumulation through a mechanism involving an IRS2-Akt-mTOR-dependent pathway (Van de Velde et al., 2011). In view of this information and our current data revealing a defect in CREB-CRE binding activity and a reduction in VEGF expression in diabetic SIECs, we examined in these cells HIF-1α dynamics under basal conditions and in response to the PKA activator MB-cAMP. A western-blotting-based technique showed a reduction in the MB-cAMP-induced elevation of HIF-1α total protein level in diabetic SIECs, as compared with corresponding control values (Fig. 4Aa). A tendency toward a decrease in *HIF-1α* mRNA level was observed in diabetic SIECs but did not reach statistical significance (control SIECs=1±0.16, diabetic SIECs=0.88±0.12). This diabetes-mediated decrease in HIF-1α protein expression could stem from a defect in phosphorylated Akt (pAkt)-mTOR-dependent signaling because this pathway has been shown to stimulate *HIF-1* translation in response to nutrient, growth factors and forskolin (Sengupta et al., 2010; Van de Velde et al., 2011). Indeed, our data depicted in Fig. 4Ab,c, which demonstrates a marked reduction in the levels of pAkt and S6 that was phosphorylated (pS6) at residues S235/236 gives credence to the aforementioned proposition. Further experiments regarding HIF-1α dynamics in diabetic SIECs revealed a diminution in the nuclear accumulation of HIF-1α in response to a 1-hour treatment with MB-cAMP (Fig. 4Ad), suggesting a possible attenuation in the HIF-1α nuclear transport mechanism.

To address the above notion of diabetes-related impairment of HIF-1α nuclear transport, we assessed the level of expression of importin α, a transport receptor that has been shown recently to be involved in the HIF-1α nuclear translocation mechanism (Ahluwalia et al., 2010). A qRT-PCR study revealed a marked reduction in importin α1 mRNA expression in diabetic SIECs (Fig. 4Ba). More importantly, we confirmed, by using western blotting and co-immunoprecipitation assays, that importin-α protein level and its binding to HIF-1α were also diminished in nuclear extracts from diabetic SIECs when compared with corresponding control values (Fig. 4Ba,b).

We then investigated whether the defect in PKA-induced CREB-CRE activation and the decrease in HIF-1α nuclear accumulation in diabetic SIECs were accompanied by a similar aberration in the binding of HIF-1 to HREs. For this purpose, an ELISA-based assay was used to quantify HIF-1α activation and binding to the HRE in nuclear extracts from control and diabetic SIECs; cobalt-chloride-treated COS-7 cells served as a positive control (Jiang et al., 1997). The wild-type HRE consensus oligonucleotide competitively inhibited HIF-1α binding to HRE in COS-7 cells, whereas the mutated consensus oligonucleotide did not. A nuclear extract derived from MB-cAMP-treated control SIECs showed a specific and time-dependent increase in HIF-1α binding to the HRE consensus; a phenomenon that was significantly diminished in diabetic SIECs and in KCREB-transfected control SIECs (KCREB is a dominant-negative CREB protein that is mutated within its DNA-binding domain). (Fig. 4Ca). To corroborate the above finding, we also measured the HIF-1α transcriptional activity using a luciferase reporter assay. For this aim, SIECs were transfected with plasmid encoding an HRE upstream of firefly luciferase (HIF-1-luc). After treatment with MB-cAMP, HIF-1-luc reporter activity in diabetic SIEC extracts, normalized against *Renilla* luciferase activity, was significantly decreased, as compared with corresponding control values (Fig. 4Cb).

Finally, to assess whether the aberration in MB-cAMP-induced activation of the nuclear transcription complex (e.g. CREB-HIF-1α and the HRE consensus) in diabetic SIECs was associated with a defect in HIF-1α binding to the HRE within the *VEGF* promoter, we performed a chromatin immunoprecipitation (ChIP) assay. Fractionated chromatin from MB-cAMP-treated control and diabetic SIECs were immunoprecipitated by using an antibody against HIF-1α or control IgG. Activation of CREB signaling by the PKA activator showed a considerable increase in the occupancy of HIF-1α on the *VEGF* promoter in control but not in diabetic SIECs (Fig. 4Cc). To provide further support for our data revealing that the association of HIF-1α with the *VEGF* promoter is impaired as a function of diabetes, a plasmid expressing the reporter gene luciferase under the control of a fragment of the human *VEGF* promoter containing the HRE was transiently transfected into control and diabetic SIECs, and then these cells were incubated with MB-cAMP for 24 hours. We found that MB-cAMP induced approximately 2.5-fold increases in luciferase activity in control SIECs that had been transfected with the reporter plasmid when compared with the untreated controls, a phenomenon that was not evident in cells of diabetic origin (Fig. 4D). Collectively, these results suggest that the diabetic state, by interfering with the CREB-CRE binding capacity, negatively alters HIF-1α dynamics and that this in turn inhibits VEGF transcriptional activity, thus resulting in the impairment of angiogenesis.

### CREM/ICER upregulation attenuates CRE-HIF-1α activation and impairs angiogenesis in diabetic SIECs

**Fig. 5.**
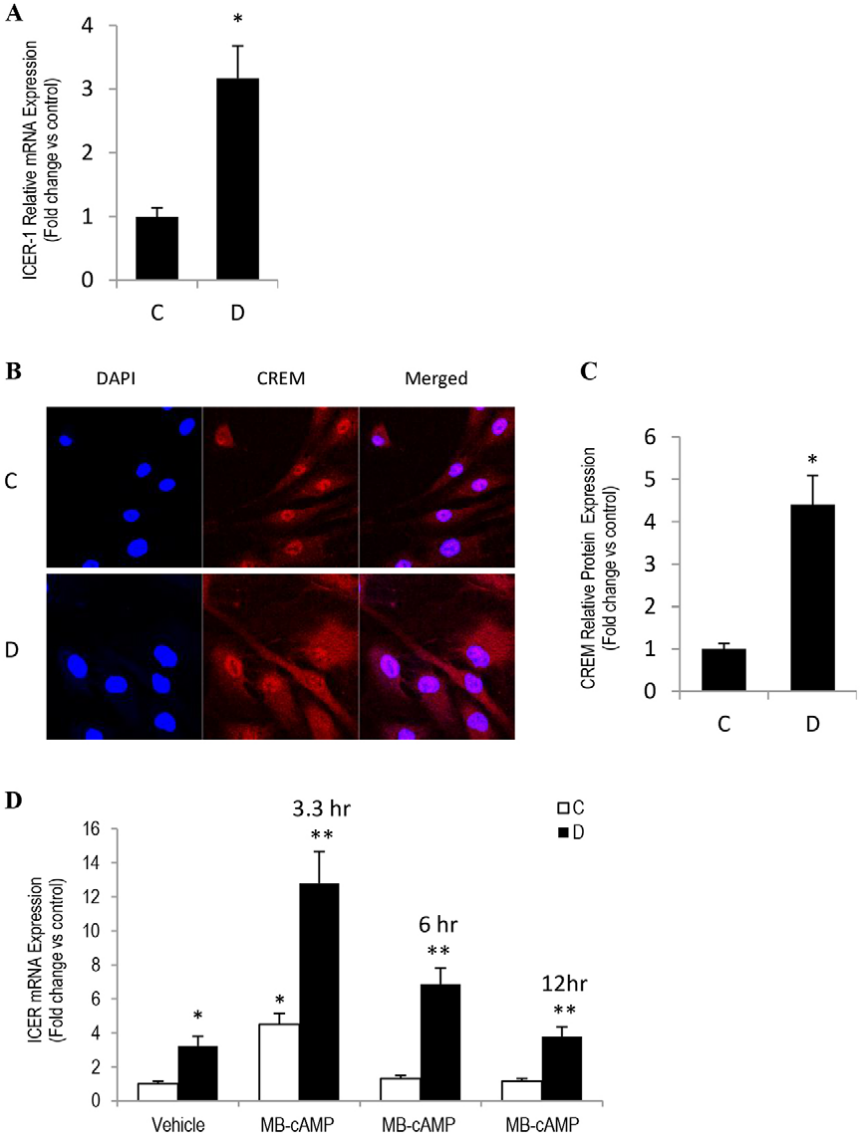
**CREM/ICER attenuated CRE-HIF-1α activation in diabetic SIECs.** (A) qRT-PCR analysis of *CREM*/*ICER1* mRNA in control and diabetic SIECs. Actin was used as loading control. (B) Representative images of immunofluorescent staining of CREM-1α (CREM) in control and diabetic SIECs. (C) Quantitative measurement of the fold change in the intensity of the CREM-1α fluorescence shown in B. (D) SIECs were incubated in the presence or absence of 100 μM of MB-cAMP for various time intervals, and then *ICER* mRNA levels were determined by using qRT-PCR. ‘C’, control; ‘D’, diabetic. Results are expressed as means±s.e.m. from three independent experiments. *Significantly different from corresponding control values at *P*≤0.05. **Significantly different from corresponding MB-cAMP-treated control values at *P*≤0.05.

CREM has been suggested to serve as a bona fide endogenous inhibitor of PKA-CREB-dependent signaling (Macho and Sassone-Corsi, 2003; Molina et al., 1993; Xie et al., 2008). It is transiently induced by cAMP-PKA agonists, and this action is followed by a suppression of CRE transcriptional activity through means of a negative-feedback mechanism (Klinger et al., 2008). Accordingly, we reasoned that an elevation in endogenous cAMP-PKA signaling activity in diabetic SIECs could elicit an upregulation of CREM/ICER expression, leading to the observed decrease in CRE and HRE transcriptional activity in these cells. Consistent with this notion, a marked increase in CREM/ICER expression was evident, both at the mRNA and protein levels (Fig. 5A–C). Moreover, we also confirmed that, in control SIECs, MB-cAMP induced a transient increase in *ICER* mRNA expression, with a maximal effect occurring 3 hours after treatment and returning to approximate basal values by 6 hours (Fig. 5D). By contrast, diabetic SIECs that were exposed to the PKA activator exhibited an exaggerated and persistent elevation in *ICER* mRNA transcription, extending for up to 12 hours after treatment (Fig. 5D). To this end, we believe that the diabetic state interferes with the negative-feedback regulation of endothelial CREB-ICER- and CREB-CREM-dependent pathway. Consequently, the excessively produced ICER/CREM proteins competitively inhibit the activation of CRE by pCREB. Indeed, our data showing that the endothelial expression of various CREB-driven angiogenic genes – including *NURR1*, *IRS2*, and more importantly, *VEGF* – were diminished as a function of diabetes (Fig. 3A,Dd) give credence to this proposition.

### ICER loss of function in diabetic SIECs restores CRE-HRE responsiveness to PKA stimulation

**Fig. 6.**
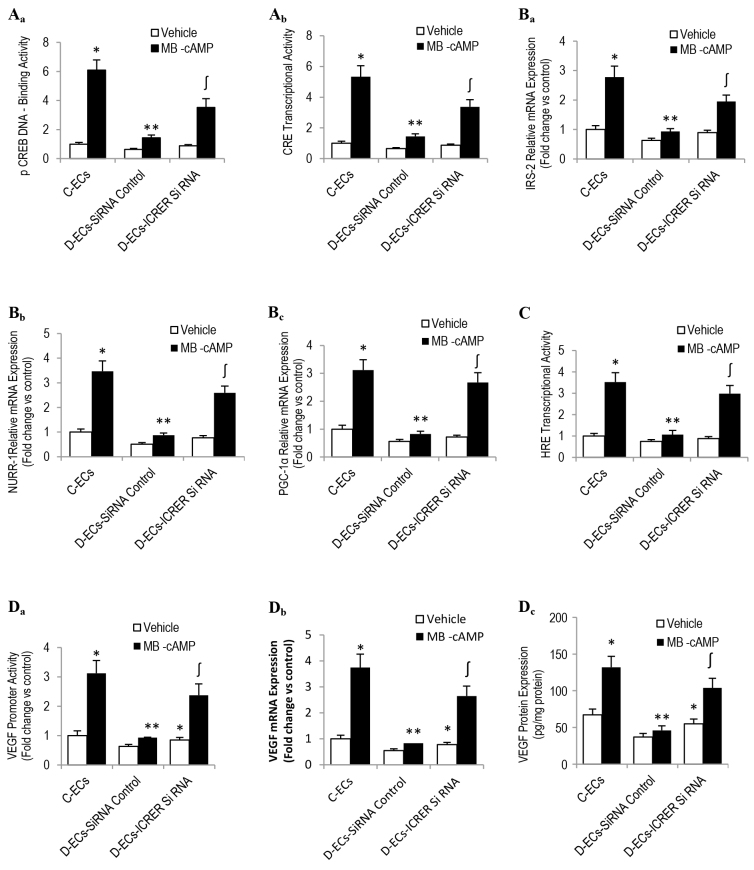
**Effects of ICER deficiency on MB-cAMP-induced activation of PKA-CREB-VEGF signaling and angiogenesis.** Diabetic SIECs were rendered ICER1 deficient (ICER1 siRNA) by using siRNA. Transfection efficiency was confirmed using TaqMan real-time PCR and western blotting. After culturing for 48 hours, cells were exposed to MB-cAMP (100 μM) for various time intervals and were then used to evaluate pCREB-DNA binding activity (Aa, MB-cAMP for 2 hours), CRE transcriptional activity (Ab, MB-cAMP for 6 hours), mRNA expression of CRE target genes (Ba-c, MB-cAMP for 2 hours), HIF-1α-HRE binding affinity (C, MB-cAMP for 2 hours), *VEGF* promoter activity (Da, MB-cAMP for 24 hours), *VEGF* mRNA (Db, MC-cAMP for 6 hours) and VEGF protein expression (Dc, MB-cAMP for 24 hours). ‘C-ECs’, control sponge implant endothelial cells; ‘D-ECs’, diabetic sponge endothelial cells. Results are expressed as means±s.e.m. for three independent experiments. *Significantly different from corresponding control values at *P*≤0.05. **Significantly different from corresponding MB-cAMP-treated control values at *P*≤0.05. ∫Significantly different from corresponding D-ECs MB-cAMP-treated siRNA control values at *P*≤0.05.

To examine whether a cause-and-effect relationship exists between the overexpression of CREM/ICER in diabetic SIECs, the aberration in angiogenic capacity and PKA-CREB-dependent signaling, we downregulated the diabetes-mediated increase in CREM/ICER level using an siRNA-based strategy; transfection efficiency was confirmed by using real-time PCR and western blotting. Our data revealed that the MB-cAMP-related enhancement in the degree of pCREB binding to DNA and in the CRE transcriptional activity was greater in diabetic SIECs harboring the siRNA against ICER/CREM than they were corresponding cells receiving only the scrambled siRNA (Fig. 6Aa,b). Importantly, this increase in the potentiating effect of MB-cAMP on CRE transcriptional activity in ICER-downregulated diabetic SIECs correlates positively with the elevation in mRNAs of a number of CRE-targeted genes, including, *NURR1*, PPARγ coactivator 1α (*PGC-1α*) and *IRS-2* (Fig. 6Ba–c). This confirms that the aforementioned strategy affected endogenous CREB-responsive genes and not simply reporter constructs.

Because the status of CRE transcriptional activity reflects, in large part, the degree of activation and binding of CREB, with the latter being involved in recruiting and facilitating HIF-1α association with HRE within the *VEGF* promoter, we assessed this sequence of events in diabetic SIECs harboring siRNA against ICER. Knocking down ICER in diabetic SIECs markedly amplified the stimulatory effect of MB-cAMP on HIF-1α signaling, as exemplified by the enhancement in HRE transcriptional activity (Fig. 6C, MB-cAMP 16 hours). More intriguingly, it also ameliorated diabetes-related defects in *VEGF* promoter responsiveness to MB-cAMP (Fig. 6Da, MB-cAMP 16 hours), as well as in the expression of this growth-promoting polypeptide, both at the mRNA (Fig. 6Db, MB-cAMP 6 hours) and protein levels (Fig. 6Dc, MB-cAMP 24 hours).

Taken together, the above findings support the concept that overexpression and persistent elevation of ICER in diabetic SIECs contribute, at least in part, to the confirmed attenuation in CRE and/or HRE transcriptional activity in these cells. Further, it also harmonizes with the notion that endothelial cells within the diabetic milieu of the wound appear to be defective in the production and, possibly, release of the most potent pro-angiogenic factor VEGF. It is worthy of note that no attempt was made in the current study to test whether the diabetic angiogenic phenotypic features can be recapitulated in control SIECs with the use of an ICER/CREM gain-of-function genetic-based strategy.

### A heightened state of oxidative stress might also contribute to CREM/ICER overexpression in diabetic SIECs

**Fig. 7.**
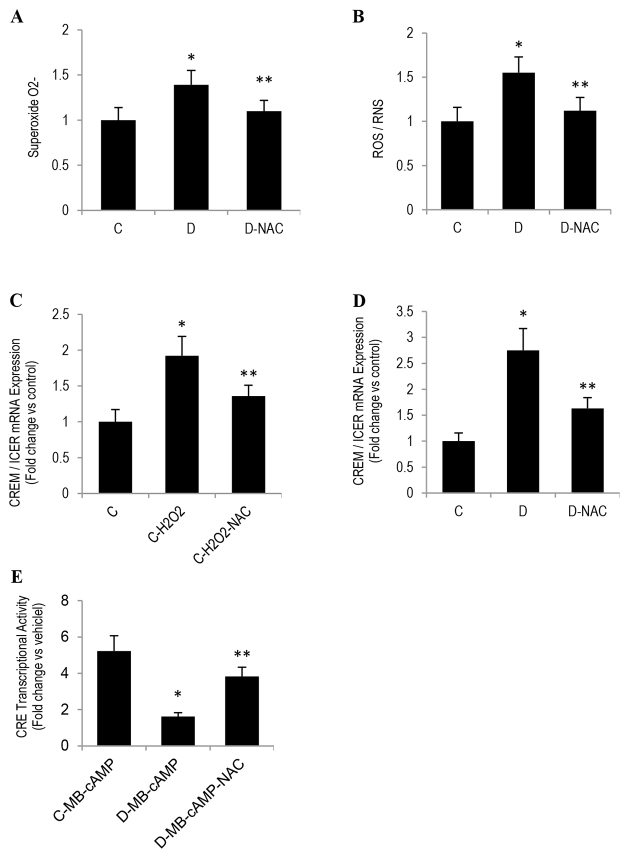
**N-acetyl cysteine (NAC) reverses oxidative-stress-induced upregulation of CREM/ICER expression in SIECs.** (A,B) Superoxide and ROS and RNS (ROS/RNS) production were measured in SIECs using the total ROS/Superoxide Detection Kit. A 24 hours, (C) control or (D) diabetic SIECs that had been treated with 150 μM H_2_O_2_ were exposed to NAC (2 mM) for 24 hours and the expression of (*ICER*) mRNA was quantified using TagMan real-time PCR. (E) SIECs were transfected with a CRE-driven luciferase reporter construct and *Renilla* luciferase control plasmid for 24 hours. After transfection, cells were exposed to MB-cAMP (100 μM) for 6 hours, and the intensity of the luciferase reactions, normalized to their *Renilla* luciferase control activity, was measured using a dual luciferase assay kit. ‘C’, control; ‘D’, diabetic. Results are expressed as means±s.e.m. for three independent experiments. *Significantly different from corresponding control values at *P*≤0.05. **Significantly different from corresponding H2O2-treated control or MB-cAMP-treated diabetic values at *P*≤0.05.

We next investigated the possible mechanism(s) responsible for diabetes-induced persistent accumulation of CREM/ICER in endothelial cells under basal conditions and in response to the PKA activator MB-cAMP. Oxidative stress was a good candidate for three reasons: (1) oxidative stress is a constant companion of endothelial dysfunction (Lähteenvuo and Rosenzweig, 2012), (2) cells of type 2 diabetes are in a constant state of oxidative stress (Bitar et al., 2013; Bitar et al., 2010), and (3) hyperglycemia and oxidized low density lipoprotein, which are elevated as a function of diabetes, appear to induce CREM/ICER expression through a mechanism involving oxidative stress (Favre et al., 2011). To test for a possible role of oxidative stress in CREM/ICER accumulation, we first assessed the spontaneous generation of oxidative stress by using a system that detects the total levels of reactive oxygen species (ROS) and superoxides. We showed that diabetic SIECs exhibited approximately 1.55-fold higher levels of ROS and reactive nitrogen species, when compared with those of control SIECs (Fig. 7A). As a positive control, SIECs were treated for 30 minutes with the ROS-inducer Pyocyanin (200 μM). Similarly, a higher level of superoxides was also evident as a function of diabetes (Fig. 7B). We then confirmed, using the pro-oxidant H_2_O_2_, that oxidative stress can indeed increase the expression of CREM/ICER in control SIECs (Fig. 7C). The most intriguing finding, however, was related to the fact that pre-incubation of diabetic SIECs or H_2_O_2_-treated control SIECs with N-acetyl cysteine ameliorated, firstly, the persistent increase in CREM/ICER level and, secondly, the impairment in CRE transcriptional activity in response to MB-cAMP (Fig. 7E).

Overall, the current data point to the possibility that a heightened state of oxidative stress in SIECs of type 2 diabetes might promote the persistent expression of CREM/ICER in response to PKA activation. This might lead to the repression of genes that contain a CRE within their promoters, thus culminating in the inhibition of both VEGF expression and angiogenesis. Obviously, further studies remain to be conducted in order to delineate the signaling pathways that link oxidative stress to the overexpression of CREM/ICER during diabetes.

## DISCUSSION

Tissue healing in response to ischemia, infarction, stroke and incisional and excisional wounds necessitates blood vessel growth, a process which appears to be attenuated as a function of diabetes (Ergul et al., 2014; Xu et al., 2012). In addition to reducing the endogenous angiogenic response, diabetes might also limit the response to exogenous intervention, such as the angiogenic growth factors delivered in clinical trials. The current study was conducted to elucidate the molecular and cellular mechanisms that contribute to impaired angiogenesis during the course of type 2 diabetes. Subcutaneous sponge implants were used to study in GK rats the various components of the neovascular response: fibrovascular invasion, cell proliferation, blood vessel morphology and density, collagen deposition and distribution, and pro- and anti-angiogenic molecules. Moreover, a focus was also drawn to the impact of the PKA-CREB-signaling axis on the pro-angiogenic properties that are essential for reparative neovascularization.

Herein, we report that diabetes impaired the *in vivo* sponge angiogenic capacity by decreasing fibrovascular invasion, capillary density, collagen deposition and VEGF expression. By contrast, the levels of TSPs, NFκB activity and expression of the pro-apoptotic protein FasL were augmented in the aforementioned diabetic sponge model of angiogenesis. An *in vitro* culturing system involving SIECs confirmed that decreased VEGF levels as a function of diabetes appear to be mediated, at least in part, by increasing cAMP to activate PKA, which in turn upregulates CREM/ICER expression to inhibit pCREB-DNA binding and to limit the transcription of *VEGF*. The functional consequence of this cascade of events was illustrated by a marked reduction in endothelial cell proliferation, migration and tube formation. A genetics-based strategy in diabetic SIECs using siRNA against CREM/ICER significantly augmented CRE-HIF-1α-VEGF-dependent signaling.

Angiogenesis, a process that occurs during the course of tissue healing and cardiac hypertrophy, appears to be altered as a function of diabetes (this work; Ergul et al., 2014). This phenomenon is regulated by a delicate balance between endogenous pro-angiogenic and anti-angiogenic molecules. Our data confirmed that in conditions such as diabetes this angiogenic balance is altered in a manner that is consistent with an over-production of the TSP and PEDF angiostatic factors and a downregulation of VEGF expression. This duality of effect of diabetes as a suppressor of VEGF and an inducer of TSPs is reminiscent of those reported previously in aged animals (Sadoun and Reed, 2003) and in cultured endothelial cells overexpressing decorin (Neill et al., 2012).

As an attempt to illustrate the molecular mechanism of the diabetes-related decrease in VEGF expression and its implications for impaired angiogenesis, we examined the PKA-CREB and HIF-1α dynamics in diabetic SIECs. This strategy harmonizes with the wealth of evidence that indicates that in a variety of cell lines, including cancer cells (e.g. SK-Hep1, PC-3; Park et al., 2011) and human umbilical vein endothelial cells (Namkoong et al., 2009; Zhang and Daaka, 2011), activation of PKA-CREB- or HIF-1α-dependent pathways increases both VEGF transcriptional activity and the formation of new blood vessels. We recognize that most of these previous studies were conducted on cells that do not resemble wound or cardiac ischemic endothelial cells because of the lack of exposure to an elaborate set of micro-environmental cues *ex vivo*. For example, the wound fluid bathing the wound tissue reflects the wound microenvironment and shapes the functional response of wound-related cells, such as endothelial cells (Drinkwater et al., 2002). To this end, the current study addressed this gap by isolating intact endothelial cells (SIECs) from the actual wound milieu using subcutaneous sponge implants, a well-established *in vivo* model of angiogenesis. We found that diabetic SIECs exhibited a significant reduction in VEGF expression both at the mRNA and protein levels. This was associated, to our surprise, with increased, rather than decreased, activity of cAMP-PKA-CREB-dependent signaling. Intriguingly, however, CREB-DNA binding and CRE transcriptional activity essential parameters for HIF-1α and VEGF formation were diminished during the course of diabetes.

Numerous studies indicate that the outcome of CRE-mediated gene expression is dictated by CREB activation and the competitive binding of several dimerized transcription factors, including activators and repressors of gene transcription (Sakamoto et al., 2011). Among the members of the CREB, CREM and ATF-1 gene families, ICER is unique in that it has the capacity to serve as an endogenous repressor of genes containing a CRE sequence within their promoters and also in that it exhibits a feature of inducibility in response to a variety of stimuli, including those that activate the cAMP-PKA-CREB network (Borlikova and Endo, 2009). Under physiological conditions, CREM/ICER induction is a transient phenomenon that allows cAMP signaling to return to the basal state (Abderrahmani et al., 2006). By contrast, prolonged or inappropriate induction of ICER can elicit pathological consequences (Abderrahmani et al., 2006). To this end, a persistent elevation of PKA activity stemming from diabetes-induced upregulation of both AC3, AC8 and downregulation of PDE3A (M.S.B. and F.A-M., unpublished observation), which are the main controllers of the temporal and spatial features of cellular cAMP production, could, by means of a positive-feedback mechanism, enhance ICER expression and consequently inhibit the transcriptional activity of CRE. Indeed, the current data demonstrating that ICER mRNA and protein expression were elevated in diabetic SIECs in connection with a reduction in a number of CRE target genes, such as *NURR1*, *IRS2* and *VEGF*, give credence to the aforementioned proposition. These findings are not unique to the diabetic SIECs as a persistent elevation of CREM/ICER and the concomitant suppression of gene transcription was also evident in other pathological conditions and cell types, such as hypercortisolemia (Shepard et al., 2005), hypercatecholemia (Lewin et al., 2009; Leopold et al., 2007) and hyperglycemia (Cho et al., 2012), in addition to angiotensin-II-treated cardiomyocytes (Ding et al., 2005) or oxidized LDL-treated insulin-secreting cells (Favre et al., 2011).

Activation of the VEGF gene can also be modulated by the total and nuclear levels of HIF-1α, a transcription factor that plays a central role in tumor progression and angiogenesis (Laughner et al., 2001). HIF-1α protein expression and activity is controlled not only by hypoxia but also by growth factors (e.g. IGF1) and cAMP- or PKA-inducing agents (Semenza, 2010; Van de Velde et al., 2011). In this context, it has been shown in a variety of cell lines (e.g. INS-1, PC-3, SK-Hep1, MDA-MB-231) that forskolin, norepinephrine or isoproterenol upregulate HIF-1α protein expression, in part through PKA-mediated activation of IRS2-AKT-mTOR-dependent signaling (Park et al., 2011; Van de Velde et al., 2011). Similarly, importin α, a transport receptor, increases HIF-1α nuclear translocation and therefore its activity in cardiac endothelial cells (Ahluwalia et al., 2010). Ensuing studies in diabetic SIECs unveiled a diminution in HIF-1α protein level in total cell lysates under basal conditions and in response to the PKA activator MB-cAMP. A greater decrease in the nuclear content of HIF-1α was evident in these cells. Others have reported a decrease in HIF-1α-dependent angiogenesis in ischemic-hindlimb models of insulin resistance and pioglitazone-treated rats (Dromparis et al., 2014). Consistent with these data, we also found that the ability of MB-cAMP to induce the activity of HRE-luc and the binding of HIF-1α to the HRE within the promoter region of *VEGF* were attenuated as a function of diabetes. A pressing question that follows these observations is how does diabetes inhibit HIF-1α expression and its translocation to the nucleus? PKA activation enhances IRS2 accumulation through CREB-CRE-dependent mechanisms (Van de Velde et al., 2011). IRS2 in turn increases HIF-1α accumulation and activity by stimulating Akt-mTOR signaling (Van de Velde et al., 2011). In this connection, exposing cells to an inhibitor of PKA (e.g. H89), CREB (e.g. overexpressing A-CREB) or mTOR activity (e.g. rapamycin) suppresses the *HIF-1α* mRNA translation (Van de Velde et al., 2011). Similar data were obtained using RNAi-mediated knockdown of IRS2 (Van de Velde et al., 2011). In line with these findings, the current study showed a reduction in diabetic SIECs IRS2 content, stemming possibly from a sustained activation of PKA and the ensuing CREM/ICER-mediated suppression of CREB-CRE transcriptional activity. This diabetes-induced IRS2 downregulation could, by means of inhibiting the Akt-mTOR axis, contribute to the observed reduction in HIF-1α total protein level. Our hypothesis is supported by the fact that the MB-cAMP-related increase in pAkt and pS6 S235/236 was attenuated in diabetic SIECs. Further experiments revealed a diminution in HIF-1α nuclear content in MB-cAMP-treated diabetic SIECs. Such a phenomenon could be a reflection of the observed decrease in the total cellular level of HIF-1α and/or a defect of importin α, an adaptor protein that binds to the nuclear localization signal and facilitates the transport of large proteins (≥40–60 kDa) into the nucleus (Lange et al., 2007; Hu et al., 2005). With regard to the last notion, we have demonstrated that the protein expression of importin α1 and its binding to HIF-1α were markedly reduced as a function of diabetes. Our findings are consistent with a recently reported aging-associated decrease in importin α in human skin fibroblasts and cardiac endothelial cells (Pujol et al., 2002; Ahluwalia et al., 2010). To this end, the dysregulation of the nuclear transport mechanism in diabetic SIECs might contribute, at least in part, to decreased HIF-1α nuclear expression and transcription, leading to reduced activation of the *VEGF* gene and henceforth impairment of angiogenesis.

An adequate level of HIF-1α and its target gene *VEGF* might necessitate reciprocal and flexible signaling between ICER and the CREB-IRS2-mTOR pathway. In diabetic SIECs harboring siRNA against ICER, MB-cAMP-induced activation of the IRS2-Akt-mTOR axis was significantly higher than that in corresponding cells that had been transfected with control siRNA. Consistent with these findings, we also demonstrated in these cells a marked increase in HIF-1α and VEGF levels. The impact of this strategy on the downstream signaling of VEGF, as well as its functional relevance to *in vitro* and *in vivo* angiogenesism, during the course of type 2 diabetes is currently under consideration in our laboratory.

**Fig. 8.**
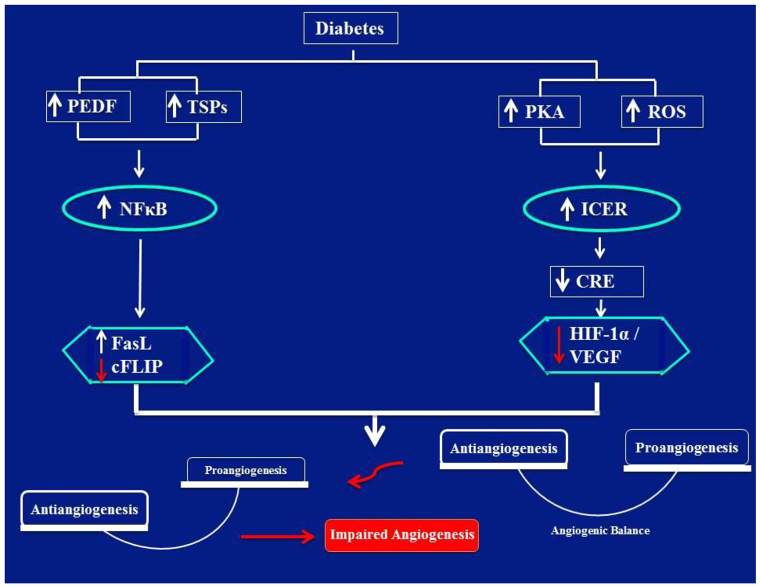
**Schematic image denoting an imbalance between pro- and anti-angiogenic signaling that culminates in impaired angiogenesis in diabetic SIECs.** Diabetes increases ROS production and enhances PKA activity, possibly by increasing AC3 and AC8 activity and/or decreasing PDE3 expression; (2) chronic activation of PKA and/or a heightened state of oxidative stress stabilizes and elicits persistent elevation of ICER; (3) excessive accumulation of ICER inhibits CRE transcriptional activity with a concomitant reduction in IRS2 levels; (4) decreased IRS2 cellular content suppresses an Akt-mTOR-mediated increase in *HIF-1α* mRNA translation; (5) reduced HIF-1α availability for HREs suppresses *VEGF* gene activity; (6) downregulation of VEGF in connection with the already existent elevation in TSP1 and/or TSP2, and NFκB impair the basic angiogenic properties; henceforth, culminating in the various forms of cardiovascular disorders and non-healing wounds.

Overall, the present data can be unified in the following model (Fig. 8): diabetes increases ROS production and enhances PKA activity, possibly by increasing AC3 and AC8 and/or decreasing PDE3 expression; (2) chronic activation of PKA and/or a heightened state of oxidative stress stabilizes and elicits persistent elevation of ICER; (3) excessive accumulation of ICER inhibits CRE transcriptional activity with a concomitant reduction in IRS2 levels; (4) decreased IRS2 cellular content suppresses an Akt-mTOR-mediated increase in *HIF-1α* mRNA translation; (5) reduced HIF-1α availability for HRE suppresses *VEGF* gene activity; (6) downregulation of VEGF in connection with the already existent elevation in TSP1 and/or TSP2 and NFκB impair the basic angiogenic properties; henceforth, culminating in the various forms of cardiovascular disorders and non-healing wounds.

## MATERIALS AND METHODS

### Animals and an *in vivo* sponge model of angiogenesis

All animal procedures were performed in accordance with the National Institutes of Health (NIH) Guidance for the Care and Use of Laboratory Animals. An established model of angiogenesis typified by subcutaneously implanted gel foam sponges was used to evaluate the *in vivo* angiogenic capacity in female GK rats (aged 12–15 months), a model for non-obese type 2 diabetes. Age- and sex-matched Wistar rats were used as a corresponding control. We have extensive experience of using GK rats to assess a number of cardiovascular- and wound-healing-related events, both *in vivo* and *in vitro* (Bitar, 2012; Bitar et al., 2013; Bitar and Al-Mulla, 2011; Bitar et al., 2010). The general procedure for gel foam sponge insertion and its suitability as an angiogenic model have been described previously by us and others (Bitar, 2000; Auerbach et al., 2000; Bradshaw et al., 2001). Briefly, sterile gel foam absorbable sponges (Pharmacia and Upjohn, Peapack, NJ) were cut into pieces and hydrated overnight at 4°C in sterile PBS. Excess PBS was removed by blotting. Animals were anesthetized by an intraperitoneal injection of ketamine and xylazine, and the dorsal surface was shaved, cleaned with alcohol and incised at the midline. Four subcutaneous pockets were made on either side of the incision (2–3 cm away from the incision) with blunt-ended forceps. One sponge was inserted into each pocket before closing the incision with 0–6 sutures (Fine Science Tools, Foster City, CA, USA). Ten days later, the rats were euthanized and the gel foam sponges were removed, washed once with PBS and then either immersion-fixed overnight in 10% buffered formalin for histochemical evaluation or frozen in OCT compound and/or liquid nitrogen for immunofluorescence microscopy or various biochemical and molecular assays.

### Quantification of an *in vivo* rate of proliferation in sponge implants

Animals with implanted gel foam sponges were given an intraperitoneal injection of BrdU at a dose of 50 mg/kg of body weight 8 hours before euthanizing. Sponges were removed, fixed in neutral buffered formalin and embedded in paraffin blocks. Tissue sections were examined for BrdU incorporation according to a previously published procedure (Muskhelishvili et al., 2003).

### Assessment of fibroblast invasion, microvascular density and collagen quality in sponge implants

Hematoxylin-eosin stained sponge sections were evaluated, by using microscopy, for fibrovascular invasion. Similarly, microvascular density was calculated in CD31-stained sponge sections using an immunofluorescence-based technique. Finally, for collagen assessment, sponge sections were stained with a Picrosirius Red, an anionic dye that differentiates collagen fiber thickness and density based on the color emitted under polarized light and captured by light microscope. Mature collagen fibers usually emit a red color (Kesler et al., 2000).

### SIECs as an *in vitro* model of angiogenesis

An overnight PBS-hydrated sponge containing endothelial cell growth supplement was inserted subcutaneously into an anesthetized rat. Eight days later, the sponges were removed aseptically and used for the isolation of endothelial cells, as described previously (Dong et al., 1997). The isolated cells were cultured under standardized conditions (37°C, 5% CO_2_) in 25-cm^2^ flasks pre-coated with 1% gelatin (type B from bovine skin, Sigma) in 20% fetal calf serum (FCS) Dulbecco’s modified Eagle’s medium (DMEM) containing 20 mM HEPES, sodium pyruvate, freshly added heparin and endothelial cell growth supplement. Confluent cells were passed routinely at a split ratio 1:3 and then cultured as described above.

### Assessment of key endothelial functions essential for angiogenesis

To assess cell proliferation, endothelial cells were seeded into a 96-well plate at a density of 1×10^4^ per well and were allowed to adhere overnight in 10% FCS-DMEM medium with endothelial cell growth supplement. After arrest by incubation in serum-free medium for 24 hours, the cells were exposed to the PKA activator MB-cAMP (BIOLOG), and the incorporation of BrdU into DNA was determined using the manufacturer’s protocol (Roche Diagnostics). Similarly, for the *in vitro* wound (migration) experiments, cultured endothelial cells were grown in 6-well plates until they reached confluence. The medium was removed; the cells were washed with PBS three times before culturing was continued in serum-free DMEM containing 0.5% bovine serum albumin (BSA) for an additional 24 hours. Thereafter, the monolayer was artificially wounded by using a pipette tip to scratch across the plate; the cells were washed with PBS to remove the detached cells and then cultured in serum-free medium in the presence of mitomyocin C (10 μg/ml) to prevent cell proliferation. The rate of wound healing was quantified according to our previously published procedure (Al-Mulla et al., 2011). Finally, a tube-formation assay was conducted by initially coating a 96-well plate with 50 μl/well growth factor reduced Matrigel (BD Biosciences), followed by incubation for 30 minutes at 37°C to allow gel polymerization. Endothelial cells (1.5×10^4^) in 100 μl of starved medium were seeded onto each Matrigel-coated well in the presence of MB-cAMP. Images of forming tubes were captured after 12 hours of incubation using a Spot-camera-equipped inverted microscope. The branching area of the tube network in the entire field of each well was calculated with ImageJ software (NIH).

### Assessment of mRNA and protein levels in sponge implants and SIECs

#### Real-time PCR for mRNA quantitation

Total RNA from cells or frozen sponge implants was extracted using Trizol reagent (Invitrogen), and the RNA integrity was verified through agarose gel electrophoresis. Approximately 1 μg of RNA was reverse transcribed (Superscript II Reverse Transcriptase Kit, Invitrogen) and amplified using the TaqMan Assay on Demand (Applied Biosystems) in a 25 μl reaction volume containing two unlabeled primers, a 6-carboxyfluorescein-labeled TaqMan MGB probe and the master mix. The amplified sequences were assessed using the ABI 7500 Prism Sequence Detection system machine. The results were expressed as mRNA levels normalized to those of *18S* or *GAPDH* in each sample.

#### Immunoprecipitation and western blot

Cells or sponge implants were sonicated on ice in radioimmunoprecipitation (RIPA) buffer containing 1% NP-40, 0.5% deoxycholate and a protease-phosphatase inhibitor cocktail (Roche Diagnostics), and the resulting homogenates were centrifuged at 15,000 ***g*** for 15 minutes at 4°C. Nuclear extracts were prepared using a nuclear isolation kit and according to the manufacturer’s instructions (Active Motif). Protein concentrations in total cell extracts or nuclear fractions were determined by using the BCA protein assay (Pierce). For immunoprecipitation, 500 μg of the supernatant protein was incubated with 20 μl of Protein A/G agarose (Santa Cruz Biotechnology) and 5 μg of antibody overnight at 4°C under constant rotation. Nonspecific IgG was used as a negative control. Immunoprecipitates were washed twice with RIPA buffer before the addition of 2× Laemmli buffer. Proteins derived from nuclear fractions, total lysates or immunoprecipitates were loaded onto an SDS-polyacrylamide gel and transferred to a PVDF membrane (Bio-Rad). The membranes were blocked and then incubated with the primary antibody diluted in 5% non-fat dry milk in TBST buffer (10 mM Tris-HCl, pH 7.5, 150 mM NaCl, 0.05% Tween 20) overnight at 4°C. After washing, the blots were incubated with the secondary antibody conjugated to horseradish peroxidase (HRP) in TBST for 1 hour at room temperature. The proteins were visualized with a Super Signal West Pico-Chemiluminescent Substrate (Pierce) according to the manufacturer’s protocol.

#### Immunofluorescence imaging

Paraffin or OCT sponge sections and coverslip paraformaldehyde-fixed SIECs were permeabilized with 0.1% Triton X-100 in PBS for 15 minutes, washed three times with PBS and incubated for 1 hour at room temperature in blocking buffer containing PBS and 2% BSA. Coverslips or sponge sections were incubated with a primary antibody overnight at 4°C, followed by washing three times with PBS and then incubating for 1 hour with the appropriate fluorescence-labeled secondary antibody. Samples were mounted in anti-fade medium with DAPI (Vector Laboratories). An LSM 510L confocal laser scanning microscope (Carl Zeiss) with Axiovert 100M was used to analyze immune-stained samples and to capture representative Images. Confocal images were converted to 8-bit grayscale. Images obtained were exported by using Axio-Vision software (Carl Zeiss) in TIFF format.

### Oxidative stress measurements

A heightened state of oxidative stress in SIECs was assessed using the total ROS/Superoxide Detection Kit (Enzo Life Sciences). Briefly, cells were incubated in a wash buffer containing 2 μM of oxidative-stress- (green) or 2 μM of superoxide-detection (orange) reagent kit. Fluorescence was measured using a fluorescence microplate reader and standard fluorescein (excitation=488 nm, emission=520 nm) and Rhodamine (excitation=550 nm, emission=610 nm) filter sets.

### Luciferase reporter assays

SIECs were transfected with either CRE-, HRE- (SA Biosciences) or pVEGF-Luc (a gift from Samuel J. Leibovich, University of Medicine and Dentistry of New Jersey, NJ, USA) or a plasmid encoding the *Renilla* luciferase under the control of the CMV promoter as an internal control. Twenty-four hours thereafter and following treatment with MB-cAMP, cells in the 96-well plate were treated with a Passive Lysis Buffer, and Firefly or *Renilla* luciferase activity was measured with a Dual Luciferase Assay Kit (Promega) and a luminometer (Promega), according to the manufacturer’s instructions.

### DNA-binding assay

An ELISA-based assay (TransAM HIF-1α, pCREB) was used to assess the interaction of HIF-1α with HRE or pCREB with CRE according to the instructions provided by the manufacturer. Briefly, nuclear extracts were incubated in a 96-well plate pre-coated with the HRE or pCREB consensus oligonucleotides. Antibodies against HIF-1α or pCREB were added to the reactions and a HRP-conjugated secondary antibody was used to quantify the HIF-1α or pCREB nuclear binding activity.

### ChIP assay

ChIP was performed using the QuikChIP assay (Imgenex) according to the manufacturer’s instructions. Protein-DNA complexes were immunoprecipitated with an antibody against HIF-1α. The PCR primers used to amplify a HIF-1α-responsive region in the *VEGF* gene −944 to −611 were the same as those reported previously (Huang et al., 2010).

### ELISA quantification of VEGF cellular content

The level of VEGF in SIECs was assessed using a commercially available kit specific for rat VEGF in cell extracts (Ray Biotech) according to the manufacturer’s instructions.

### siRNA transfection

The expression of CREM/ICER in SIECs of type 2 diabetes was genetically downregulated by using siRNA oligonucleotides. The sequences were designed and synthesized by Qiagen. The day before transfection, cells were seeded at a density of 1.75×10^5^ cells/well in a 6-well plate in complete DMEM medium. The next day, cells were washed once with OptiMEM medium (Gibco) and then overlaid with 800 μl of OptiMEM medium. Optimum silencing efficiency was obtained by adding 18 μl of 20 μM siRNA to 142 μl of 37°C OptiMEM medium, incubated at room temperature for 15 minutes, and then Oligofectamine mixture (Invitrogen) was added according to the manufacturer’s instructions (8 μl Oligofectamine + 32 μl of OptiMEM medium, incubated for 5 minutes at room temperature). Complexes were added to the cells and after 4 hours of incubation in the CO_2_ incubator, 500 μl of DMEM containing 30% FCS was added to each well. Following overnight incubation, the cells were washed with PBS and incubated in DMEM medium. Efficiency of the knockdown of CREM/ICER was verified by using real-time PCR or western blotting

### Statistical analysis

Data are expressed as the means±s.e.m. Comparisons were made by using a two-tailed paired Student’s *t*-test or a one-way analysis of variance followed by Bonferroni post hoc test. A level of *P*≤0.05 was considered to be significant.
